# Composition of the Sand Fly Fauna in Khash County, Southeast Iran

**DOI:** 10.1673/031.012.13201

**Published:** 2012-11-10

**Authors:** Hamid Kassiri, Ezatoeddin Javadian

**Affiliations:** ^1^ School of Health, Ahvaz Jundishapur University of Medical Sciences, Ahvaz, Iran; ^2^ School of Health, Tehran University of Medical Sciences, Tehran, Iran

## Abstract

Sand flies (Diptera: Psychodidae: Phlebotominae) are the biological vectors of leishmaniasis all around the world. In 1997, sand flies were collected in 14 cities and villages of Khash County in southeastern Iran, using 848 sticky traps (castor oil-coated white papers 20 × 30 cm). In this study, a total of 4673 sand flies, with 25.23% females and 74.77% males, were collected and identified to species mainly from mountainous areas. The 21 species of sand flies belonged to the genus *Phlebotomus* (nine species) and the genus *Sergentomyia* (12 species). The following 14 species were reported for the first time in Khash County: *P. papatasi, P. bergeroti, P. eleanorae, P. halepensis, P. major, P. mesghali, S. hodgsoni, S. mervynae, S. dreyfussi, S. iranica, S. theodori, S. africana, S. clydei,* and *S. christophersi.* The composition of species in Khash County is similar to other parts of Iran. However, the dominance of *P. kazeruni* in Khash County may suggest that this species should be considered as a potential vector in the region of Khash.

## Introduction

Three forms of leishmaniasis including zoonotic cutaneous leishmaniasis, anthroponotic cutaneous leishmaniasis, and visceral leishmaniasis are public health problems in Iran and its neighboring countries such as Afghanistan and Pakistan. Zoonotic cutaneous leishmaniasis is the most abundant and endemic form of the disease in Iran, particularly in the East and Southeast foci. The city of Khash is near Chabahar, Mirjaveh, and Zahedan, which are the South and Southeast leishmaniasis foci in Iran (Yaghoobi-Ershadi and Javadian 2006; Motazedian 2008; Jahanifard et al. 2009; Azizi and Fekri 2010). In total, 231 cutaneous lieshmaniasis cases were reported from Chabahar during 1997–1998. The seasonal dispersal during 1997–1998 was reported as follows: 6.5, 19.9, 31.2, and 42.4% in spring, summer, fall, and winter, respectively. The incidence rate of the disease was 5.8, 6.4, 28.11, 18.54, and 25.21 per million during 1995–1999. A survey of the Phlebotomine species in Khash County appears to be an important step in the control of leishmaniasis.

Sand flies (Diptera: Psychodidae: Phlebotominae) are the biological vectors of leishmaniasis. The first comprehensive entomological study on sand flies of Iran was done by Mesgali (1960), who reported 12 species belonging to the genus *Phlebotomus* and 11 species for the genus *Sergentomyia.* Later, Javadian and Mesghali (1974) reported 42 species of Phlebotomine sand flies in Iran. More recently, Rassi et al. (2004) showed that the fauna of Iran includes 44 confirmed species and 10 unconfirmed, as reported by the latest Iranian Phlebotomine sand fly faunistic studies (Seyedi-Rashti and Nadim 1992; Kasiri et al. 2000; Rassi and Hanafi 2006; Rasoolian et al. 2007; Jahanifard et al. 2009; Azizi and Fekri 2010). Finally, Kassiri et al. (2000) have proposed a checklist of Iran sand flies including 54 species. In Iran and in the Old World, *P. papatasi* is recognized as the main vector of leishmaniasis to humans (Rassi et al. 2004; Jahanifard et al. 2009).

The objectives of the present study were to determine sand fly species diversity, relative population abundance, and sex ratio. These data provide basic epidemiologic information for vector population control programs to reduce the incidence of zoonotic cutaneous leishmaniasis in the region.

## Materials and Methods

### Study area

The investigation was carried out in 1997 in Khash County, which is located in Sistan-Baluchistan Province, Southeast Iran (28° 14′ N, 61° 12′ E, 1394 m.a.s.l.). Khash County has a dry climate with a total rainfall of 154 mm per year. Diurnal and seasonal temperature variations range from 44 °C in summer to 18.40 °C in winter. The relative humidity ranges from 11.9% in September to 73.4% in January. Khash County has a population of about 130,000 people (Taher-Shamsi and Moussavi 2003).

### Sand fly collection and identification

Sand fly collections were conducted in 14 cities and villages of Khash County: Khash, Nookabad, Eskalabad, Baluchabad, Sabzgaz, Gezo, Irandegan, Dadkan, Reeis, Pawel, Sangan, Karvandar, Gunich, and Tamin. The sand flies were collected using 848 sticky traps (castor oil-coated 20 × 30 cm strips of white paper), mainly in mountains and plains areas during the early summer of 1997. The traps were installed after sunset and were collected before the following sunrise. Only one replicate (14 samplings) was performed during the early summer per location because of local problems in the area of the study.

**Table 1.**
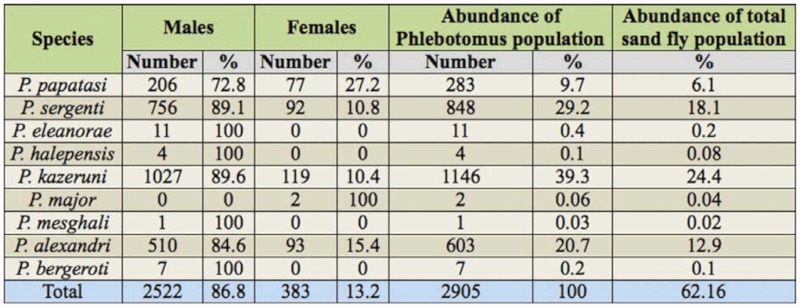
Abundance of *Phlebotomus* species of Khash County.

**Table 2.**
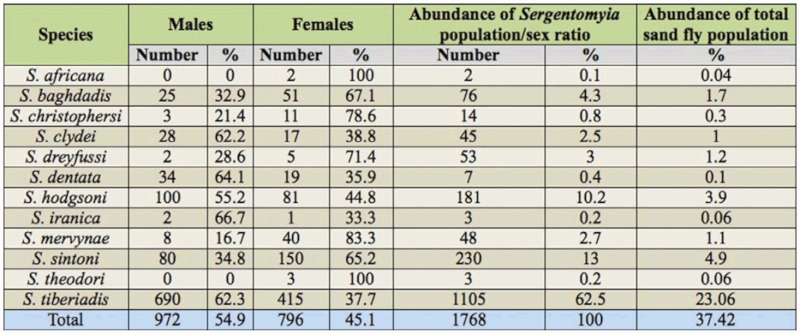
Abundance of *Sergentomyia* species of Khash County.

Sand flies were removed from the traps, rinsed in acetone, and preserved in 70% ethanol. All specimens were sexed and the head capsule, ventral side facing up, mouth parts and end parts of male and females, including male and female genitalia, were separated. They were placed in Puri's clearing medium for one week, which is sufficient to clear, and were dry mounted on microscope slides, including male terminalia, spermatheca, pharynx armature, and buccal armature. Finally, they were mounted on microscope slides, using Puri's medium (Smart et al. 1965). The species were identified using the keys of Lewis (1982), Nadim and Javadian (1976), and Seyedi-Rashti and Nadim (1992).

## Results

A total of 4673 sand flies, of which 25.23% were females and 74.77% were males, were collected from the outdoor locations. Of the 21 species of sand flies identified, nine belonged to the genus *Phlebotomus* and 12 belonged to the genus *Sergentomyia.* The results are summarized in Tables 1 and 2. The most abundant species were *P. kazeruni* and *S. tiberiadis,* representing 39.3% of *Phlebotomus spp.* and 62.5% of *Sergentomyia spp.,* respectively. The 21 species that were reported for the first time from Khash County can be seen in Table 3.

The sex ratios (number of males/females × 100) showed that the traps were more attractive for males, in particular for *P. kazeruni, P. sergenti, P. alexandri, P. papatasi,* and *S. tiberiadis* (Table 1). All details of data are presented in the Tables 1 and 2.

**Table 3.**
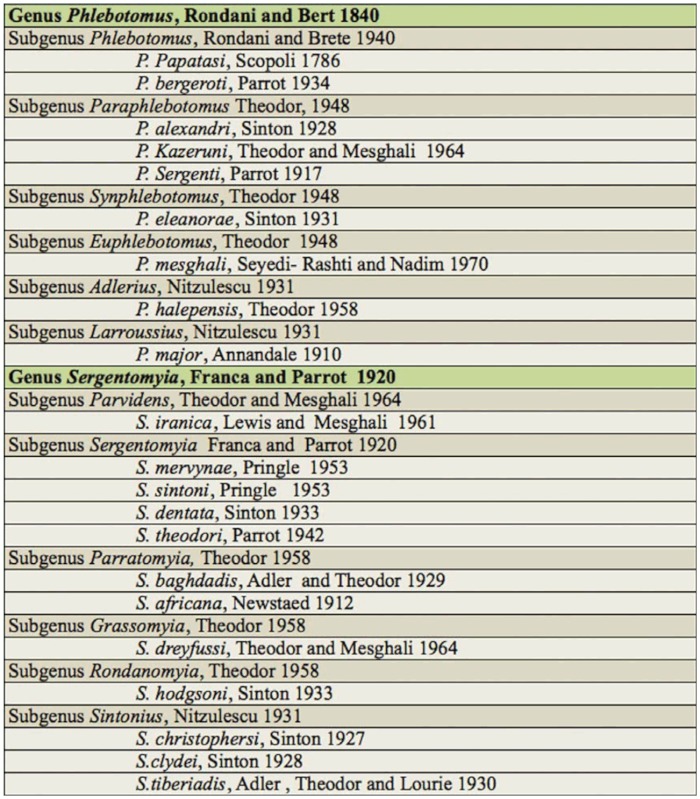
List of Phlebotominae of Khash County.

## Discussion

In the current study, nine species of *Phlebotomus* and 12 species of *Sergentomyia* were collected. *Phlebotomus kazeruni* was the most abundant sand fly species. This result is different from similar surveys that found *P. papatasi* to be the predominant species. However, the Khash area is a mountainous region and *P. kazeruni* is mostly found in mountains. Nevertheless, regarding species composition, the results of this study are similar to other studies carried out in nearby areas. The results of Kakarsulemankhel (2004, 2010) surveys carried out in Baluchistan (Pakistan) showed the presence of seven species of *Phlebotomus,* among which four were collected in the present study: *P.* *papatasi, P. bergeroti, P. sergenti,* and *P. alexandri.* Among the 14 species of *Sergentomyia* collected in Baluchistan, four were also collected in the current study: *S. baghdadis, S. clydei, S. tiberiadis,* and *S. hodgsoni.*

The list of sand flies species collected in the present study compared with the results of Kassiri and Javadian (2000, 2011) in Chabahar, Southeast Iran, show that seven species of the genus *Phlebotomus: P. papatasi, P. sergenti, P. alexandri, P. kazeruni, P. bergeroti, P. mesghali,* and *P. eleanorae;* and 11 species of the genus *Sergentomyia: S. clydei, S. sintoni, S. tiberiadis, S. baghdadis, S. hodgsoni, S. dentata, S. africana, S. dreyfussi, S. mervynae, S. iranica,* and *S. chiristophersi,* were found in both regions. The results of this study differed from the results of the survey of Chabahar (Kassiri and Javadian 2000) in regards to *P. papatasi* and *P. sergenti,* as more females were collected than males of these two species in this study. However, the results of both studies were similar in regards to *P. alexandri,* as 90% of individuals of this species collected in this study were male. This difference is likely due to ecological conditions, as well as geographical and weather differences. The Khash area is a mountainous dry region, and Chabahar is located in the plains and is a more humid region.

Another study carried out in Arsanjan County, south of Iran, collected eight species of *Phlebotomus (P. papatasi, P. sergenti, P. alexandri, P. mongolensis, P. andrejevi, P. tobbi, P. keshishiani,* and *P. halepensis)* and four species of *Sergentomyia (S. sintoni, S. dentata, S. theodori,* and *S. clydei)* (Rassi et al. 2004). Among the 12 species identified in Arsanjan County, nine species were reported in the current study. The most common species was *P. papatasi. Phlebotomus kazeruni* was not reported in Arsanjan County. Apart from different ecological conditions (Khash is mountainous and Arsanjan County is a plains area), the method of sand fly collections were different. In the current study, the sand flies were collected outdoors with sticky traps from cracks, crevices, and holes of mountains, and in the Arsanjan study the sand flies were collected from rodent nests using sticky traps.

In Shahrood region, East of Iran, Abaii et al. (2007) reported only three species: *P. papatasi, P. caucasicus,* and *S. sintoni,* with *P. papatasi* being the predominant species. The species *P. caucasicus* was not collected in our survey. Sex ratios for *P. papatasi* and *S. sintoni* in Ahmad Abad and Bekran villages showed a greater attraction of the traps for females (Abaii et al. 2007), contrary to what was found in our study. In southern Iran, Motazedian et al. (2006) collected only *P. papatasi.* However, the very poor sand fly species diversity of both studies (both Abaii et al. (2007) and Motazedian et al. (2006)) may be related to the parasitology (rather than faunistic) objectives of the surveys.

In Shahreza County, central Iran, Motovali Emamia and Yazdi (2008) collected nine *Phlebotomus* species: *P. papatasi, P. major, P. sergenti, P. mongolensis, P. caucasicus, P. keshishiani, P. ansarii, P. longiductus,* and *P. halepensis,* and three *Sergentomyia* species: *S. Sintoni, S. dentata,* and *S. pawlowskyi.* The same species, except *P. keshishiani, P. ansarii, P. longiductus,* and *S. pawlowskyi,* were all collected in the present study.

In South of Tehran, Iran, Nekouie et al. (2006) reported three species: *P. papatasi, P. sergenti,* and *P. caucasicus,* with *P. papatasi* being dominant in Abardejeh.

In Kuhpayeh district, central Iran, the following species were found in the study of Abdoli et al. (2007): *P. papatasi, P. sergenti, P. caucasicus, P. mongolensis, P. alexandri, P. ansarii, P. major, P. kandelakii, S. sintoni, S. dentata,* and *S. pawlowskyi,* with *P. sergenti* as the dominant species. The species *P. papatasi, P. sergenti, P. alexandri, P. major, S. sintoni,* and *S. dentata* were also reported in our study.

In Qom, central Iran, Farzin-Nia and HanafiBojd (2007) collected *P. sergenti, P. major, P. alexandri, P. kandelakii, P. tobbi, P. brevis, P.(Adlerius) sp., P. halepensis, S. pawlowskyi,* and *S. theodori.* The absence of *P. papatasi* in the Qom survey was puzzling (Farzin-Nia and Hanafi-Bojd 2007). Again, *P. sergenti, P.* *major, P. alexandri, P. halepensis,* and *S. theodori* were also identified in our study.

In Damghan district of Semnan province (central Iran), Azni et al. (2010) reported *P. papatasi, P. caucasicus, P. sergenti, P. alexandri, P. ansarii, S. sintoni,* and *S. sumbarica.* In Ilam, West Iran, Javadian et al. (1997) collected 11 species from the genus *Phlebotomus: P. papatasi, P. sergenti, P. alexandri, P. major, P. tobbi, P. kandelakii, P. perfiliewi, P. balcanicus, P. halepensis, P. nadimi, P. ilami,* and 11 species from the genus *Sergentomyia: S. sintoni, S. dentata, S. antennata, S. theodori, S. mervynae, S. pawlowskyi, S. africana, S. clydei, S. tiberiadis, S. iranica,* and *S. squamipleuris.* About half of these species were found in the current survey: *P. papatasi, P. sergenti, P. alexandri, P. major, P. halepensis, S. sintoni, S. dentata, S. theodori, S. mervynae, S. africana, S. clydei, S. tiberiadis,* and *S. iranica.*

In Jask, Southern Iran, Azizi and Fekri (2010) identified eight species of sand fly, three from the genus *Phlebotomus: P. papatasi, P. major,* and *P. salehi,* and five species from the genus *Sergentomyia: S. sintoni, S. theodori, S. clydei, S. tiberiadis,* and *S. dentata.* All these species with the exception of *P. salehi* were reported in the current study.

In another study in Shiraz, in South Iran, 10 sand flies species were included on the list, three species of *Phlebotomus: P. papatasi, P. tobbi,* and *P. sergenti,* and seven species of *Sergentomyia: S. sintoni, S. theodori, S. clydei, S. dentata, S. palestinensis, S. mervynae,* and *S. sogdiana* (Rasoolian 2007). The authors also reported the presence of the subgenus *Grassomyia* of *Sergentomyia.* Most of the species found in Shiraz were also found in the current study.

The sand flies species reported from Khash County have already been reported from other regions of Iran. However, our results show that the abundance of species varies greatly in the different areas. The most abundant species in the current study, *P. kazeruni,* has never been found as the dominant species in any other study. The most abundant species in the other parts of Iran is *P. papatasi.* The abundance of *P. kazeruni* is an important finding that may have epidemiological consequences. This variation may be due to several factors such as the attractiveness of the traps, the environment, and the climatic conditions of capture. In addition, the sex ratio not only varies among the different species, but also between the different areas. Finally, the species diversity found in the current study is in an agreement with the results previously found in Chabahar region (Kassiri and Javadian 2000, 2011).

## Conclusion

The sand fly species distribution reported by our survey in Khash County is comparable to what was found in other parts of Iran, and included species from the two genera *Phlebotomus* and *Sergentomyia.* However, the dominance of *P. kazeruni* in Khash County is a new finding and may have epidemiological consequences if this species appears to be a potential vector in the region of Khash (Hanaf et al. 2007). Consequently, the results of this study, in particular for the relative abundances of the different sand flies species, show that different and uncommon leishmaniasis transmission cycles are possible in different areas of Iran.
